# Track Geometry Prediction Using Three-Dimensional Recurrent Neural Network-Based Models Cross-Functionally Co-Simulated with BIM

**DOI:** 10.3390/s23010391

**Published:** 2022-12-30

**Authors:** Jessada Sresakoolchai, Sakdirat Kaewunruen

**Affiliations:** Department of Civil Engineering, School of Engineering, University of Birmingham, Birmingham B15 2TT, UK

**Keywords:** track geometry prediction, recurrent neural network, long short-term memory, gated recurrent unit, attention, building information modeling, railway, asset management, co-simulation, digital twins

## Abstract

Railway track maintenance plays an important role in enabling safe, reliable, and seamless train operations and passenger comfort. Due to the increasing rail transportation, rolling stocks tend to run faster and the load tends to increase continuously. As a result, the track deteriorates quicker, and maintenance needs to be performed more frequently. However, more frequent maintenance activities do not guarantee a better overall performance of the railway system. It is crucial for rail infrastructure managers to optimize predictive and preventative maintenance. This study is the world’s first to develop deep machine learning models using three-dimensional recurrent neural network-based co-simulation models to predict track geometry parameters in the next year. Different recurrent neural network-based techniques are used to develop predictive models. In addition, a building information modeling (BIM) model is developed to integrate and cross-functionally co-simulate the track geometry measurement with the prediction for predictive and preventative maintenance purposes. From the study, the developed BIM models can be used to exchange information for predictive maintenance. Machine learning models provide the average R^2^ of 0.95 and the average mean absolute error of 0.56 mm. The insightful breakthrough demonstrates the potential of machine learning and BIM for predictive maintenance, which can promote the safety and cost effectiveness of railway maintenance.

## 1. Introduction

Due to the growth of the world economy, transportation demand tends to increase continuously [1]. This results in the increasing impact load that makes the track deteriorates rapidly [2] not only railway track but also other infrastructures such as overhead infrastructure [3]. Maintenance needs to be performed efficiently. Too little maintenance result in deteriorated track. At the same time, too much maintenance results in the exceed unnecessary maintenance cost. Therefore, proper maintenance is a goal. To be able to perform railway track maintenance properly, a tool to allow the current condition of the track is required. There are many criteria used to evaluate the condition of tracks. Track geometry is a criterion used to evaluate the quality of the track. The track with good quality results in smooth operation, safe service, and more passenger comfort. Track geometry needs to be maintained in a good condition. Normally, track geometry can be measured regularly using different methods. One of the popular methods is the use of track geometry cars (TGC). TGC is used to collect track geometry parameters and then they will be used to calculate the track quality index (TQI) based on the standards of each country. To maintain track geometry, different approaches can be used such as corrective maintenance, preventive maintenance, and predictive maintenance. Corrective maintenance is used to correct when problems take place. Therefore, this approach is not effective and requires high cost because problems may disturb operation and heavy maintenance normally requires a higher cost than light maintenance. In the case of track geometry, corrective maintenance can be used when any track geometry parameters exceed the defined values. Preventive maintenance is the planned maintenance to keep tracks in a good condition although track geometry parameters do not exceed the standard limit. Therefore, preventive maintenance has the benefit of keeping track in the good condition. However, the cost effectiveness might not be good if the maintenance plan is not performed efficiently. Another approach is predictive maintenance which is a data-driven approach. This technique is used to evaluate the condition of tracks and predict the condition in the coming period. This allows planners to be able to predict the future conditions of tracks and plan the maintenance effectively. However, predicting track geometry is complex and non-linear due to many factors.

This study aims to develop an information management platform for railway maintenance for being a data exchange platform to perform maintenance activities in the railway industry. At the same time, deep machine learning models will be developed to predict track geometry parameters for predictive maintenance purposes. This will allow railway operators to be able to know the current and future conditions of tracks based on track geometry parameters. The contribution of the study is expected to support the decision-making of railway maintenance agencies for better actions in the aspect of railway maintenance. Machine learning models which are used to develop predictive models are three-dimensional recurrent neural network-based models consisting of vanilla or traditional recurrent neural network (RNN), long short-term memory (LSTM), gated recurrent unit (GRU), and attention.

Track geometry parameters consist of seven parameters which are superelevation, longitudinal level of both rails, alignment of both rails, gauge, and twist. From the literature review, most previous studies apply machine learning to predict deterioration rates, track a quality index, and track geometry parameters. The deterioration rate and track quality index are the summary of calculations from seven track geometry parameters so each parameter cannot be predicted. This study aims to develop machine learning models to predict each track geometry parameter because they can be used to calculate the deterioration rate and track the quality index further and maintenance planning can be done more effectively because maintenance activities can be planned based on each parameter. More detail is presented in Section 3.

Because this study uses time-series data based on distances, times, and maintenance activities to predict track geometry parameters, an ability or platform that can store information is required. Therefore, this study develops a building information modeling (BIM) model to store, manage, and exchange information. Normally, BIM models are used in the design and construction stages. When the construction completes, BIM models are no longer used. However, the maintenance and operation stages are the longest stages in railway projects. Applying the BIM concept to the maintenance and operation of railway projects brings significant benefits because the amount of information in these stages of railway projects is enormous. It is predicted that results from the study will present the potential of BIM and machine learning integration in railway maintenance. The novelties of the study are this study purposes a workflow to cross-functionally co-simulate BIM with machine learning, which is the highest BIM maturity (BIM Level 5) [4], in order to predict track geometry parameters and there has never been a study focusing on this topic. This can develop automated track maintenance in the railway industry. In addition, using three-dimensional recurrent neural network-based models to predict track geometry parameters is also another novelty of the study because other studies use different features to predict them. In summary, the integration between BIM and machine learning can provide many benefits. First, it supports automating design and analysis tasks. Machine learning can be used to analyze BIM models and extract useful information such as identifying issues in different phases of projects, optimizing the performance of asset management, and performing predictive maintenance. Second, it enhances collaboration and communication by linking information between different stakeholders. Last, BIM can be used to facilitate data management. Machine learning is suitable to manage and analyze the large amount of data generated during the BIM process, such as design documents, construction schedules, and maintenance records. This can help improve the accuracy and efficiency of data management and decision-making.

## 2. Exploration of Track Geometry Measurement and Prediction

Track geometry can be measured using different methods such as linear displacement transducers, tachometers, laser optics [5,6], and track gauges [7]. Normally, track geometry parameters are measured every foot [5]. Track geometry parameters consist of superelevation, longitudinal level of both rails, alignment of both rails, gauge, and twist [8]. To evaluate the track quality, indexes are calculated based on seven-track geometry parameters. Each country has different standards and calculation methods. For example, an average standard deviation of seven track geometry parameters is popularly used in many countries such as the United Kingdom and Australia [9]. Examples of standard responses based on track geometry are shown in Table 1 and the definition of response is shown in Table 2.

Besides using TGCs, sensors can also be used to measure track geometry. In 2002, Network Rail applied unattended geometry measurement systems (UGMS) which were installed on regular rolling stock to measure track geometry. Two accelerometers and transducers were used as sensors. However, they could not measure all track geometry parameters. They could only measure vertical profiles [11]. Ágh [12] studied the relationship between track geometry irregularity and axle box acceleration (ABA). In the study, longitudinal level and alignment were measured using decoloring of chord offset. The measurement was done every 0.25 m. The frequency of the measurement was 300 Hz. It was found that the correlation between vertical ABA and track geometry was 0.63. In the study, it was found that ABA could be used to predict longitudinal level and alignment, but it could not be used to predict twist because the correlation was too weak. Li et al. [13] tried to find the relationship between track geometry and vehicle response. They applied a neural network to develop predictive models for vehicle responses such as vertical and lateral wheel loads. The performance of the model was acceptable. Tsunashima et al. [14] estimated track geometry using car-body vibration. They applied Kalman Filter to transform data. The error from the proposed method was 3 mm. However, it was not clear how they calculate track geometry or which parameters they considered. These studies aimed to estimate current track geometry using different values.

For the prediction, Lee et al. [15] applied a support vector machine (SVM) to develop a machine learning model to predict TQI in terms of alignment and longitudinal level. The limitation of the study was not every track geometry parameter was considered. In addition, compared to other deep learning techniques, SVM is not suitable for the non-linear problem and the training time is long. Hu and Liu [16] also applied SVM to predict track degradation. They focused on three defects which were longitudinal level, superelevation, and dip. They tried to predict amplitudes of defects. Inputs of the models were track class, traffic volume, and time interval. The average accuracy of their model was 0.81. Guler [8] applied a neural network to develop a model to predict deterioration. Inputs were track structure, traffic characteristics, track layout, environmental factors, track geometry, and maintenance and renewal data. Data were collected from 2009–2011. The number of samples was 820. The average R^2^ is 0.77. Soleimanmeigouni et al. [17] used field data to develop a machine learning model using binary logistic regression to predict longitudinal levels. The period of data collection was January 2015 to July 2018. The length of the considered section was about 200 m. Inputs of the models were speed, TQI, defect, and types of assets. The prediction of the models consisted of two classes which were the occurrence of defects. They found that the sensitivity of the model is 0.89. Machine learning is also used to detect railway defects such as corrugation [18] which the performance was satisfying. Besides the mentioned literature, machine learning and mathematical models were used in different areas such as supply chain [19], railway design [20], inspection scheduling [21], and cost management [22]. The summary of related studies is shown in Table 3.

From the literature review, it can be seen that there are research gaps in this topic. First, there has not been a study predicting all seven track geometry parameters. Most studies focused on some parameters only which could not provide a comprehensive ability to predict track geometry and maintenance activities for each defect. Inputs or features of machine learning models can be more explored to find new potential in track geometry prediction. Deep learning has not been used to develop predictive models. Other minor limitations were the limited timeframe of data collection and section length which were too high that might not practical in realistic maintenance. Therefore, this study tries to fill research gaps by trying to predict every track geometry parameter using RNN-based models which are deep learning techniques. In addition, this study uses field data collected by track geometry cars during 2016–2019 with a section of 30 km that provide an abundance of data for machine learning model development. It is expected that the performance of models will be satisfied and improve the overall railway maintenance aspect.

## 3. Exploration of Track Geometry and BIM Application

BIM has been widely used in the architecture, engineering, and construction (AEC) Industry while it has not been popular in the railway industry. However, in recent years, the application of BIM in the railway industry during the design and construction processes is booming [23]. The advantages of BIM can be beneficial to the railway industry because of visualization, detailed drawings, document and information management, cost management, construction scheduling, and crash detection. It was found that using BIM can reduce up to 40% unnecessary costs, improve the accuracy of cost estimation by 3% compared to the traditional method, and reduce cost estimation time by up to 80%. In total, the construction cost can be reduced by 10% by using BIM, and construction time can be reduced by 7% [24].

BIM can be categorized into three levels based on the comprehensiveness of its application. In this study, 6D BIM level 3 which integrated project schedule, cost and quantity, and maintenance aspects is a goal to achieve. A definition of BIM level 3 which is the ultimate goal of BIM application is a single BIM model that is shared by all stakeholders at any time. However, achieving this goal is not easy. The application of BIM in the railway industry needs a high-level decision and takes a long time to be effective. Crossrail in the United Kingdom applied BIM in 2007 however it was effective in 2019 [25]. However, many countries aim to make BIM mandatory for public projects such as the United Kingdom in 2016 and France in 2017. Examples of railway projects that applied BIM in the design and construction phases were Project Mälarbanan in Sweden, Schuman-Josaphat Tunnel in Belgium, Crossrail (Elisabeth Line) in the United Kingdom, and ONCF 40 electrical substations in Morocco. Although BIM starts being applied in the railway industry, it is mostly limited to design and construction.

Société Nationale des Chemins de Fer Français (SNCF, Saint-Denis, France) is one of the first departments that applied BIM in railway maintenance. They used reverse engineering to create BIM models for existing projects and used them for predictive maintenance. The pilot project was started in 2015. They stated even though it was the very early stage but they believe in the potential of BIM [26].

From the literature review, it can be seen that most railway projects only apply BIM in design and construction. If BIM can be applied in the following stages of projects, it will be highly beneficial to the railway industry because the operation and maintenance phases are the longest phases of railway projects. Information occurring in the phases is enormous. An ability to utilize the information will help improve the efficiency of asset management. Therefore, this study aims to apply BIM to railway maintenance integrated with machine learning. It is expected that this approach can support decision-making in the early stage and its potential can be extended when more information is added to the BIM model. The developed concept of integrated BIM and machine learning can solve significant problems in the railway industry where the severity of rail defects and location cannot be determined properly, resulting in excessive additional track inspection.

## 4. Data Characteristics and Processing

Data used in this study are from two sources. First, track geometry was collected using TGCs in 2016–2019. The frequency of data collection is one foot. The length of the section is 30 km. Collected raw data consists of location, track number, superelevation, radius, longitudinal level of both rails, alignment of both rails, gauge, inclinations of both rails, test speed, a maximum speed of the track, and twist. However, some values will be removed because they are not used in this study. Outstanding values will be locations and seven track geometry parameters which are superelevation, longitudinal of both rails, alignment of both rails, gauge, and twist. Second, maintenance data was collected from maintenance reports. Maintenance reports consist of seven activities which are (1) tamping, leveling, and alignment, (2) rail grinding, (3) ballast cleaning, (4) sleeper replacement, (5) rail replacement, (6) fastening replacement, and (7) ballast unloading. Maintenance activities are matched which track geometry parameters to develop machine learning models later.

**Figure 1:**
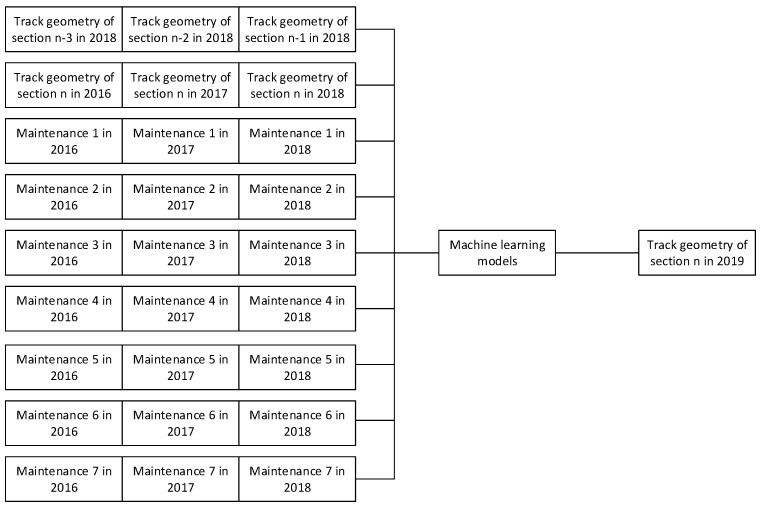
Inputs of machine learning models.

Raw data are processed to feed into machine learning models. In this study, Visual Basic for Applications (VBA) is used to process data. Data used to train machine learning models consists of three time-series data based on distances, times, and maintenance activities. Data for 2016–2019 is available. Therefore, the aim is to predict seven track geometry parameters in 2019. To do this, data from 2016–2018 are used. For time-series data based on distances, parameters of previous track sections in 2018 are used as features. For time-series data based on time, parameters of the same section in 2016–2018 are used as features. For time-series data based on maintenance activities, maintenance activities in 2016–2018 are used as binary features. If that maintenance activity was performed in that year, it will 1. Otherwise, it will be 0. There are seven maintenance activities in maintenance reports which are (1) tamping, leveling, and alignment, (2) rail grinding, (3) ballast cleaning, (4) sleeper replacement, (5) rail replacement, (6) fastening replacement, and (7) ballast unloading. The number of samples is 14,538 which will be split with the proportion of 70/30 to be training and testing data. In total, nine time-series data are used as features to train machine learning models which are shown in Figure 1 when section n represents an interesting section.

## 5. Machine Learning Model Development

Machine learning techniques used in the study are RNN, LSTM, GRU, and attention. They are RNN-based models. Inputs or features used to train models are described in Section 4. The features are stacked with each other before being fed into machine learning models. there are nine layers of features fed into models. Each feature is 1D data or time-series data. Based on the characteristics of the data, RNN-based models are suitable because the data are time-series, and the sequence of data is not very long. Therefore, RNN-based models should provide a good result. Other machine learning models are preliminarily tested; however, the performance is not as good as RNN-based models. Therefore, this study will focus on RNN-based models. a brief about each model is explained as follows.

RNN is the most fundamental of other RNN-based models. It has memory units to process sequential data which is different from normal neural networks. Therefore, features are processed in multiple units or nodes. RNN can output single or multiple outputs based on its architecture. In this study, predictions are track geometry parameters in the target year so it requires only one output. RNN has a limitation when the length of sequences is too long or more than a thousand. It suffers from a vanishing gradient according to its calculation process. The equation of RNN can be shown in (1) where $h^{n}$ is the hidden state at time *n*, $x^{n}$ is the input at time *n*, and θ is the function of the RNN. Examples of RNN applications are language processing and financial application [27].

(1) $$h^{t}=f\left(h^{t-1},x^{t};\theta \right)$$

LSTM is developed to solve the vanishing gradient in RNN. It solves the issue by including three types of gates in the architecture, input gate, output gate, and forget gate. The forget gate has a function to filter out unnecessary or meaningless data. Therefore, the model does not need to memorize all data and has a better performance. The equation of LSTM is shown in (2)–(4) where $i^{t}$ is the input gate function, $f^{t}$ is the forget gate function, $o^{t}$ is the output gate function, σ is the sigmoid function, $w^{x}$ is the weight of gate *x*, $h^{n}$ is the hidden state at time *n*, $x^{n}$ is the input at time *n*, and $b^{x}$ is the biases of gate *x*. Examples of LSTM application are language processing and weather forecast [28].

(2) $$i^{t}=\sigma \left(w^{i}\left[h^{t-1},x^{t}\right]+b^{i}\right)$$

(3) $$f^{t}=\sigma \left(w^{f}\left[h^{t-1},x^{t}\right]+b^{f}\right)$$

(4) $$o^{t}=\sigma \left(w^{o}\left[h^{t-1},x^{t}\right]+b^{o}\right)$$

GRU is more advanced than LSTM. It contains two gates which are update gates and reset gates. Compared to LSTM, the update gates are a combination between the input gate and forget gate. Therefore, the complexity of an architecture is reduced, and the model can train faster. Reset gates are used to determine how much data should be forgotten. Moreover, when the sequence of data is not very long, GRU tends to perform better than LSTM. The equation of GRU is shown in (5)–(8) where $z^{t}$ is the update gate vector, σ is the sigmoid function, $w^{n}$ is the weight of vector *n*, $x^{t}$ is the input vector, $h^{n}$ is the hidden state at time *n*, $b^{n}$ is the biases of gate *n*, $r^{t}$ is the reset gate vector, ${\hat{h}}_{t}$ is the candidate activation vector, and ∅ is the hyperbolic tangent function. Examples of GRU applications are stock price prediction [29] and time-series forecast [30].

(5) $$z^{t}=\sigma \left(w^{z}x^{t}+u^{z}h^{t-1}+b^{z}\right)$$

(6) $$r^{t}=\sigma \left(w^{r}x^{t}+u^{r}h^{t-1}+b^{r}\right)$$

(7) $${\hat{h}}_{t}=\emptyset \left(w^{h}x^{t}+u^{h}\left(r^{t}\times h^{t-1}\right)+b^{h}\right)$$

(8) $$h^{t}=z^{t}\times {\hat{h}}_{t}+\left(1-z^{t}\right)\times h^{t-1}$$

Attention is one of the latest RNN-based models. It has sub-neural networks to determine the influence of data and their significance. Data are screened before they are trained. Therefore, the architecture of the attention is similar to vanilla RNN but there is a loop to screen the data first. This is done by using the softmax function.

From the brief about each model, although some models are the improved version of another model, it cannot be guaranteed which model provides the best performance. Therefore, models have to be tested for the model section. To test machine learning models, mean absolute error (MAE) is used as the main criterion because it is simple to translate and conform to the requirement of the industry. R^2^ is also presented to demonstrate the relationship between true and predicted values.

To find the best possible architecture of each model, hyperparameter tuning is used. In this study, grid search is used for hyperparameter tuning. Hyperparameters of each model which are tuned are shown in Table 4. Each parameter affects the performance of the models in some ways. Therefore, combinations of different parameters have to be explored to find the most suitable combination. Examples of hyperparameters’ effects are the number of nodes and layers directly affecting the complexity or nonlinearity of model architecture, batch size affecting the processing time and accuracy of prediction, or activation function affecting how data are processed when sent through layers.

## 6. BIM Model Development

To develop a BIM model, Autodesk Civil 3D and Ferrovia are used. The BIM model is created based on the drawing of the project. This study aims to develop a 6D BIM model. Sequences of creation are explained as follows.

**Figure 2:**
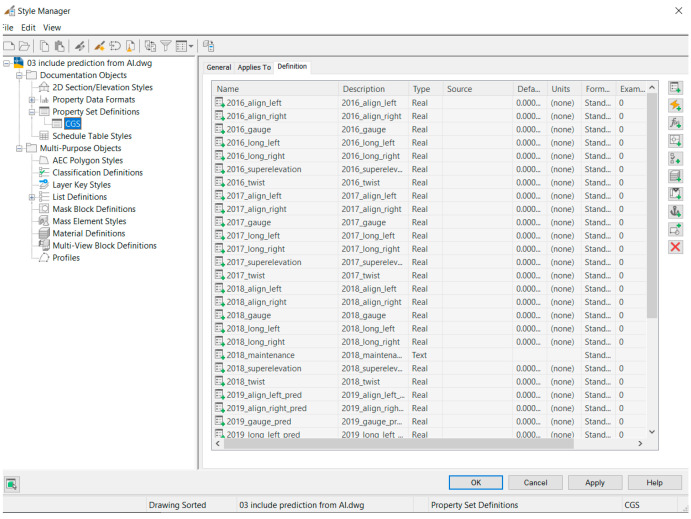
Property set definitions.

**Figure 3:**
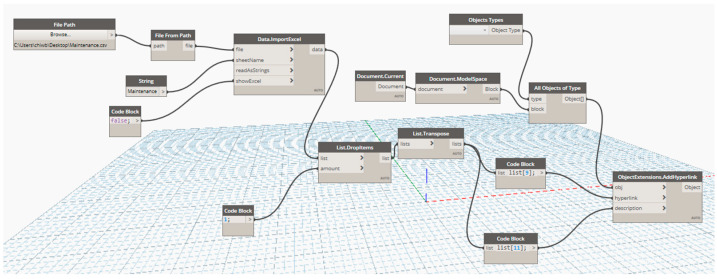
Dynamo for information exchange.

**Figure 4:**
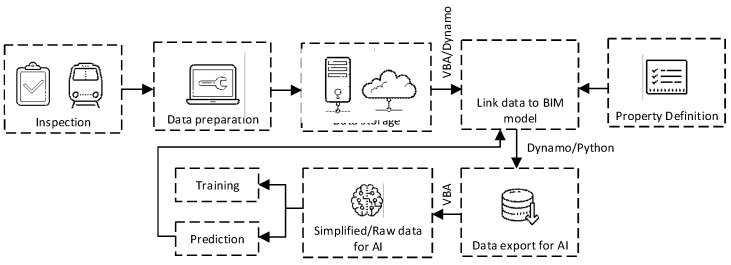
BIM and machine learning integration workflow.

The process starts by importing google map using Ferrovia. In the process, elevation data and a raster image can be imported. Elevation data will be used later when a profile is designed. The raster image is used to see the topography of the area. Then, Digital Terrain Model (DTM) is created using the imported elevation data. Triangles, boundaries, or contours can be created depending on the purpose of the application. This study mainly uses triangles. The next step is creating alignments. In the software, design speed and gauge can be defined to make sure that the design conformed to the defined standard. In this step, drawings of the project are used to re-create the alignment of the project. Sample lines are used to section the alignment. This study develops the BIM model for maintenance purposes. Therefore, the size of the sections needs to be related to the maintenance. The size of the sections is defined to be 2 m sections. When the alignments are designed, the profiles are created based on the drawings of the project. The next step is creating cross-sections of the railway line. This step is critical because it defines the railway structure and is used to calculate the bill of quantity. After that, a 3D model can be created based on defined cross-sections. The outcome of the model is multiple solid 3D that can be exported to other BIM software for further applications. Making solid 3D that can contain information for BIM applications, it can be done by defining property set definitions as shown in Figure 2. In this study, parameters used as features for machine learning models, maintenance responses in the current year, predicted track geometry parameters, and maintenance responses in the next year are added. The data can be stored in the BIM model itself. However, if the amount of data is too big, the size of the BIM model will be also big which may be obstructed the application. Therefore, in the case of big data, the data can be stored in the cloud or offline repository. These approaches are suggested because there is no limitation in terms of the data size. This can be merged with the BIM model using a hyperlink to store the link to the data or location in the offline repository. Information exchange between the BIM model and machine learning can be done using Autodesk Dynamo and VBA as shown in Figure 3. Dynamo is used to manage the BIM model including the data export and import while VBA is used to process data. Dynamo has some limitations regarding complicated processing. When they are used together, data management and exchange are seamless. The integration between the BIM model and machine learning can be done by using the developed workflow as shown in Figure 4.

Until this step, the 3D BIM model has been created and is ready to be integrated with information. For the 4D model, it includes the cost and quantity. Ferrovia has a built-in function for quantity takeoff. The Bill of quantity can be exported as a spreadsheet file and used to calculate cost estimation. To develop a 5D model, 3D solid objects in Civil 3D can be exported as Industry Foundation Classes (IFC). Ferrovia also has a function to do this. These objects can be imported to Autodesk Navisworks for project scheduling. Last, to develop a 6D BIM model from the 5D BIM model, the maintenance information mentioned can be integrated into the BIM model through the property set definitions as mentioned. Now, the 6D BIM model is available for integration with machine learning models.

## 7. Result and Discussion

### 7.1. Machine Learning Model Development

From machine learning model development and hyperparameter tuning, the best performance to predict each track geometry based on MAE can be shown in Table 5. Other indicators are also included to demonstrate the performance of models for a clearer view. R^2^ is presented to demonstrate the correlation between the true and predicted values. Maximum absolute error is presented to demonstrate the boundary of error. This value is compared to the track construction tolerance to make sure that the predictions can be used in reality. If the maximum absolute error exceeds the track construction tolerance, the prediction is not reliable because it may affect the operation safety although the MAE is small and R^2^ is high.

From the table, it can be seen that the MAEs for all parameters are less than 1.5 mm. which is small compared to their original values. For R^2^, they are higher than 0.8 in all cases so it can be concluded that the true values and predicted values are correlated, or the developed machine learning models can provide prediction with reliability. Based on the MAEs, attention performs best in predicting superelevation, and GRU performs best in predicting longitudinal levels. Other than that, LSTM performs best in predicting other track parameters. Hyperparameters of each model are fixed and used to predict every track geometry parameter. the above table presents the models which perform the best in predicting each track geometry parameter. Therefore, four models are developed in total. It can be seen that RNN cannot perform best in any parameter prediction. This can be inferred that RNN is the traditional model that was developed a long time ago. There are some limitations in the model while other models have been developed in some ways based on the vanilla RNN. In addition, it can be seen that LSTM provides the lowest MAEs from four out of seven parameters even though GRU and attention are further developed by the concept. This indicates that it cannot be concluded that the more advanced models always provide better performance than the less advanced models. It is based on the characteristic of data and models have to be tested before conclusion and application. The authors have tested other neural network models and found that the performances of the RNN-based models provide the best performance. For optimization, the crucial aspect is the completeness, availability, and variety of the data. This is because the optimization requires varied data to improve the performances of the models. Therefore, if the data are limited, the efficiency of the optimization will be worse.

It can be seen that the maximum absolute errors of all parameters are less than the track construction tolerance based on Australian Rail Track Corporation LTD [31]. The maintenance criteria shown in Table 1 are not used because the track construction tolerance is stricter and the railway industry must keep the track condition in the good condition as much as possible because safety is the priority. Therefore, the ability to predict accurate values is also crucial.

**Figure 5:**
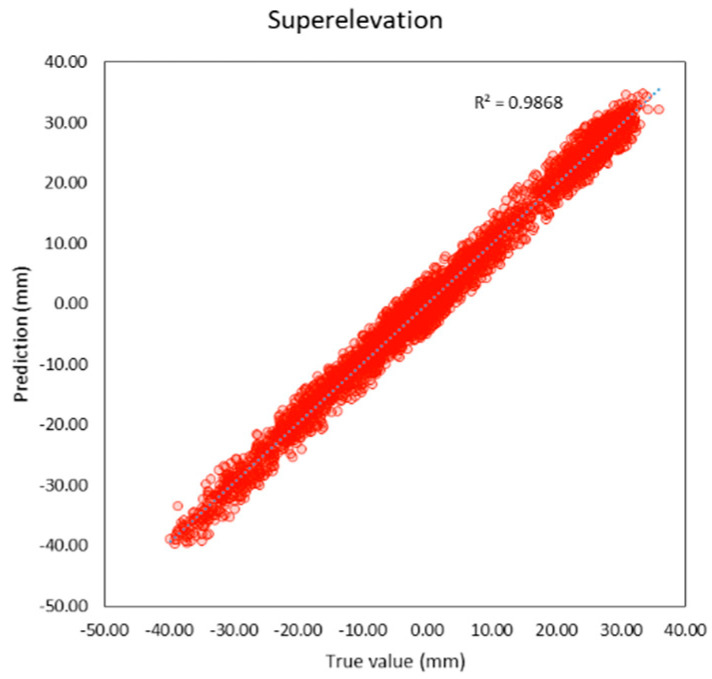
Correlation between true and predicted values of superelevation.

**Figure 6:**
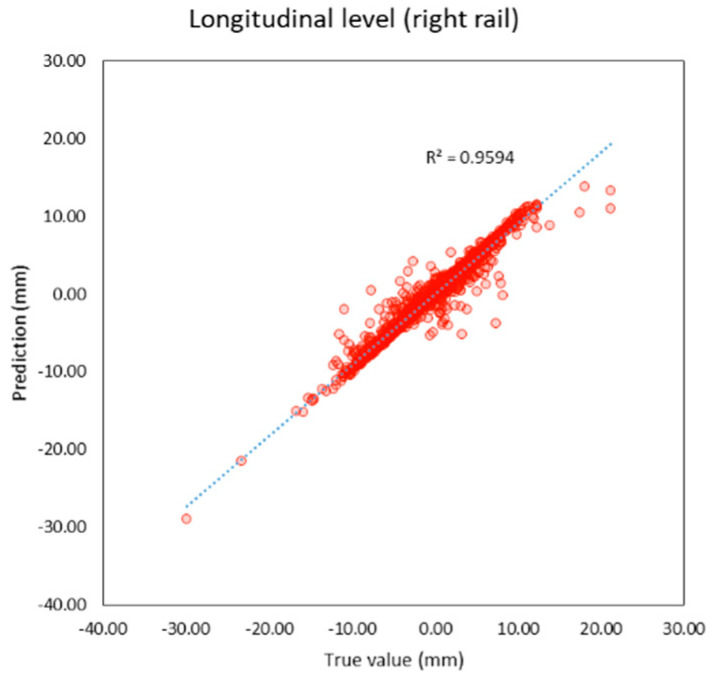
Correlation between true and predicted values of longitudinal level (right rail).

**Figure 7:**
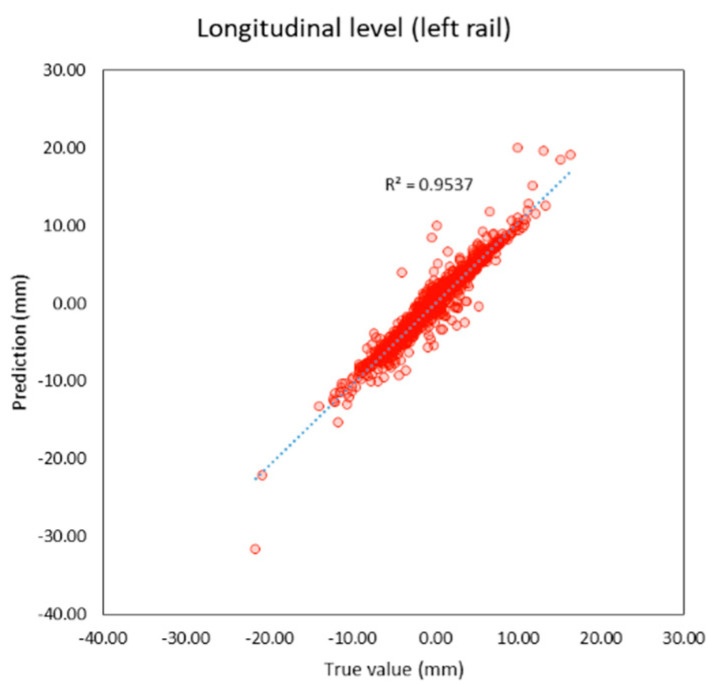
Correlation between true and predicted values of longitudinal level (left rail).

**Figure 8:**
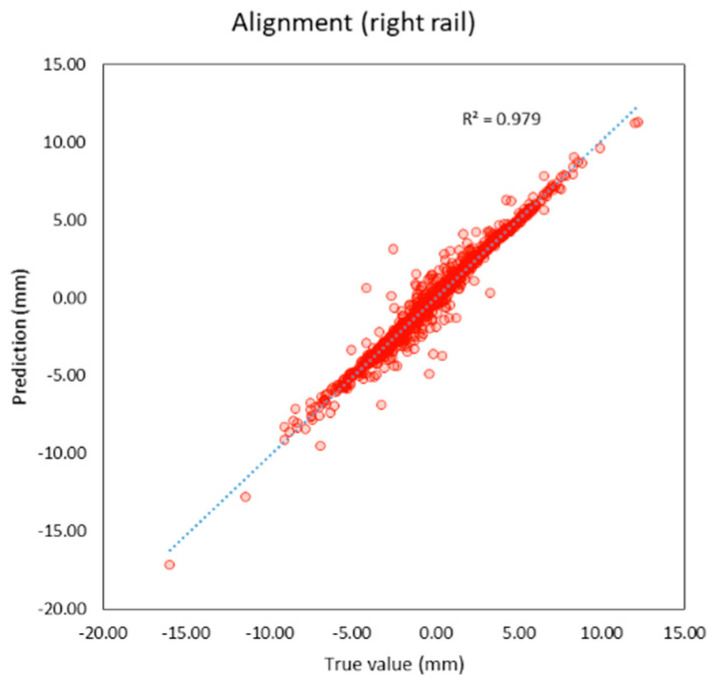
Correlation between true and predicted values of alignment (right rail).

**Figure 9:**
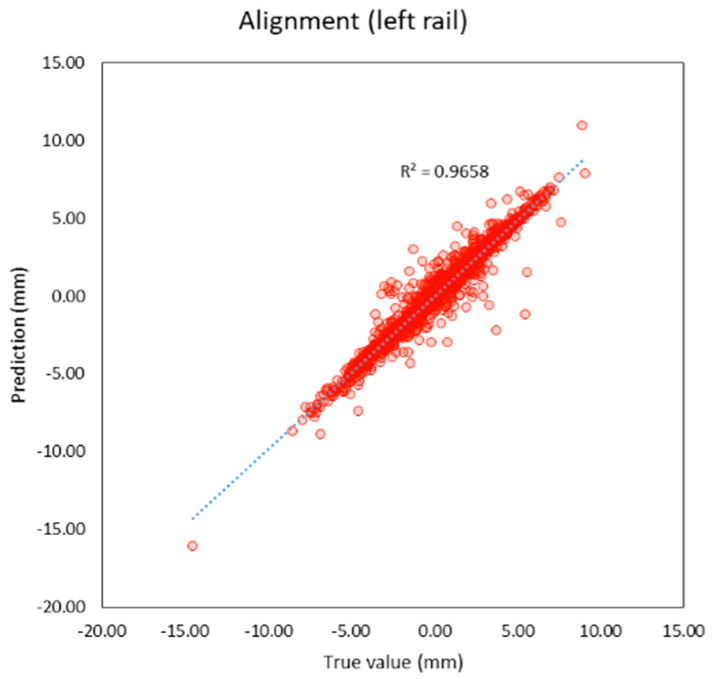
Correlation between true and predicted values of alignment (left rail).

**Figure 10:**
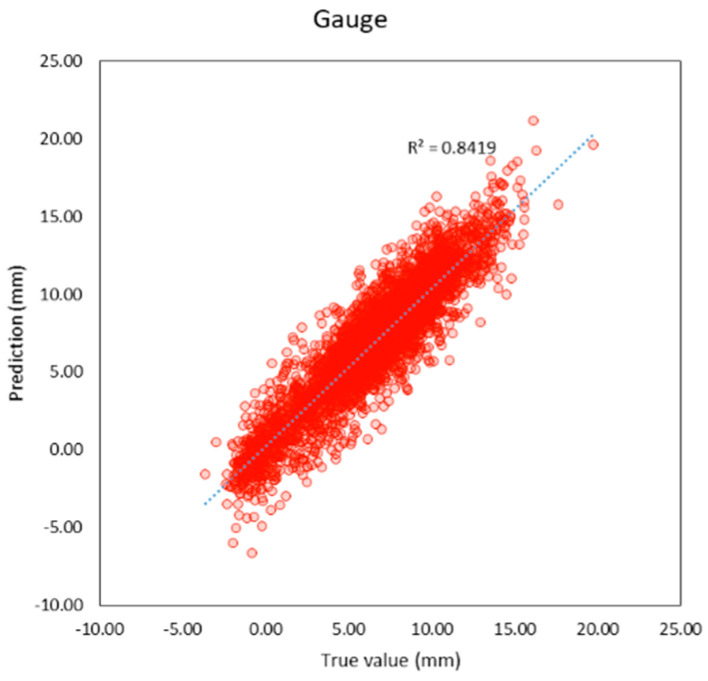
Correlation between true and predicted values of gauge.

**Figure 11:**
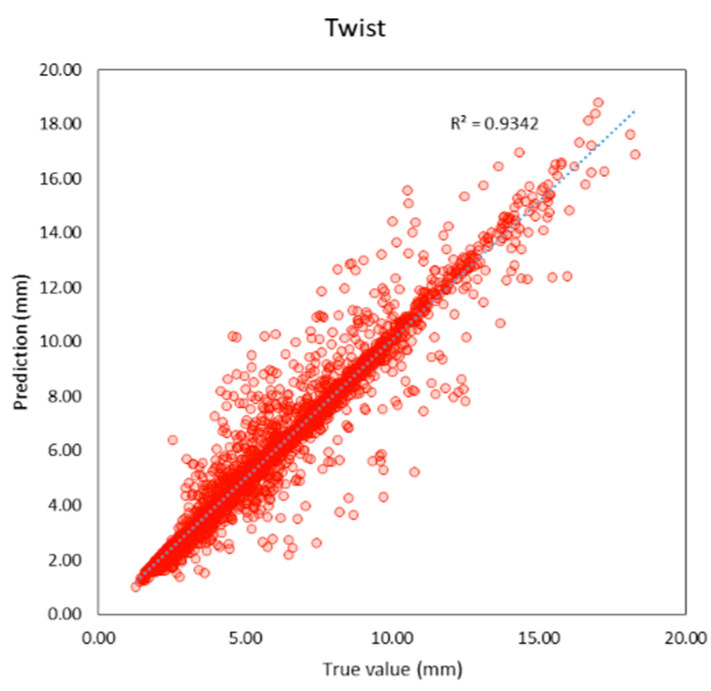
Correlation between true and predicted values of twist.

Compared to previous studies as shown in t, both individual and average R^2^ of this study are higher than previous studies. The average R^2^ of this study is 0.95 and the average MAE is 0.56 mm. which is lower than previous studies as well. It can be inferred that machine learning models in this study provide better performance because this study applies deep learning techniques that previous studies did not, and the features of the models are more suitable. R^2^ and the correlation between the true and predicted value can be shown in Figure 5, Figure 6, Figure 7, Figure 8, Figure 9, Figure 10 and Figure 11.

**Figure 12:**
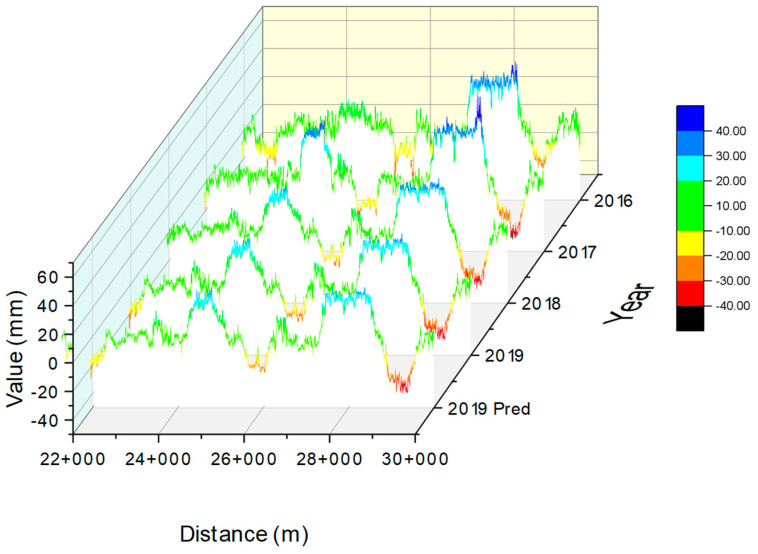
A 3D plot presenting time-series superelevation.

An example of a time-series plot is shown in Figure 12. The plot presents the superelevation in each year including the prediction from KM 22+000 to 30+000. It can be seen the true value of superelevation in 2019 is similar to the predicted superelevation. Therefore, it can be concluded that the developed machine learning models are reliable.

From grid search, hyperparameters that provide the best performance of LSTM, GRU, and attention are present in Table 6. A limitation of the study that should be noted is this study applies time-series data with three-dimension data, so the model required data with this characteristic and completeness. However, if the data are not available, non-time series data can also be used with an easy modification of the model to support non-time series data that is similar to traditional studies.

### 7.2. BIM Model Development and Integration with Machine Learning

**Figure 13:**
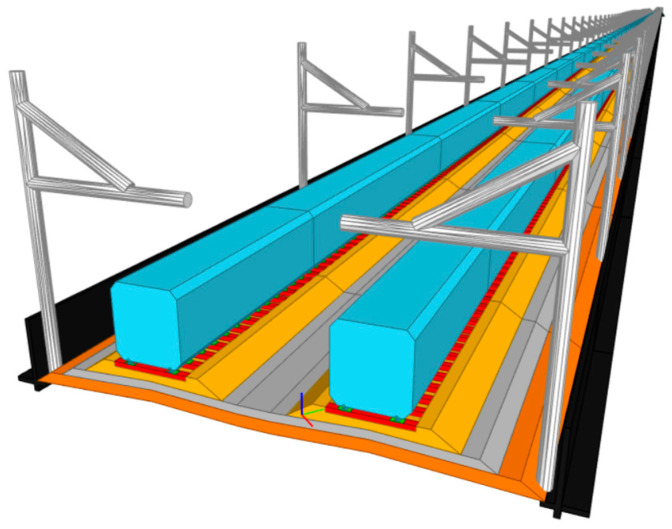
**A** 3D BIM model.

**Figure 14:**
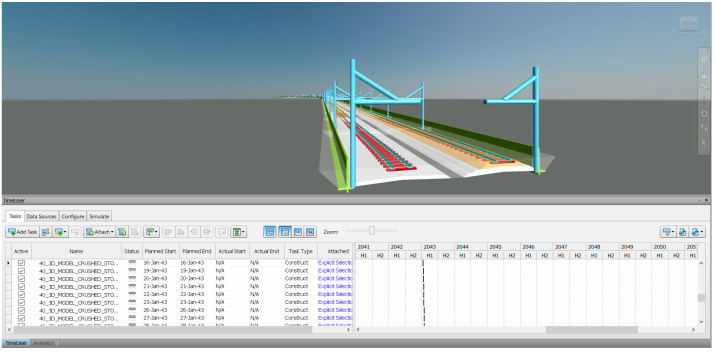
Railway BIM model imported to Navisworks for scheduling.

In Section 6, the workflow of the 6D BIM model is presented. The outcome is shown in Figure 13. Any components of the track structure can be included based on the required detail. From the figure, sleepers, baseplates, foundations, and retaining walls are included. However, more detail and components required more memory and computational power to use the BIM model. the size of sections and the number of 3D solid objects also affect the required memory and computational power. As mentioned, each 3D solid object can be calculated for quantity and used to prepare the bill of quantity to achieve the 4D BIM model. The 3D solid objects can be exported to Navisworks to prepare the project schedule in terms of construction schedules or maintenance schedules as shown in Figure 14 to achieve the 5D BIM model.

**Figure 15:**
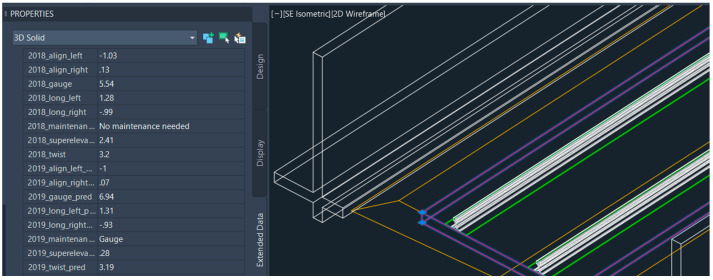
Information integrated into the BIM model.

For the 6D BIM model, the information is integrated with the BIM model using the workflow shown in Figure 3. Information integrated with the BIM model can be shown in Figure 15. The information included are features or inputs for machine learning models and results from the prediction including the maintenance activities that are required in each track section.

The developed 6D BIM model can be integrated with machine learning seamlessly and data exchange can be done automatically. The benefit of the developed BIM model, machine learning models, and workflow is they can be used together to predict each track geometry parameter and inform required maintenance responses to keep tracks in a good condition. Therefore, railway maintenance can be done more efficiently because it conforms to the predictive maintenance concept.

## 8. Application in Real Situations

The developed concept, BIM model, and machine learning models in this study can be used to predict track geometry parameters in the coming year. The prediction ability will improve the maintenance efficiency of the railway maintenance in the aspect of predictive maintenance. Railway operators can use TGCs to measure track geometry parameters. Then, collect data can be integrated with BIM models which are used as data management platforms. BIM models can store data and information. Stored data are in time-series data form. Then, they can be used to predict future track geometry. Besides preparing maintenance responses based on the measurement in the current year, railway operators can prepare, or schedule future maintenance responses based on predictions. Predictions can also be used to predict the following track geometry parameters. However, the sequence of data in this study is not long enough to test the performance of this concept. This should be further studied in the next step of the study. The developed BIM model can be used for data management along the project life cycle for asset management. This will improve the efficiency of BIM applications which are normally used in the design and construction stages only. It is believed that using BIM for the whole project life cycle will improve the overall asset management in different aspects such as cost, quality, availability, maintainability, and reliability.

## 9. Conclusions

This study aims to integrate BIM and machine learning to predict track geometry parameters which consist of superelevation, longitudinal level of both rails, alignment of both rails, gauge, and twist. The study applies RNN-based machine learning models which are RNN, LSTM, GRU, and attention to predict the parameter. Features of machine learning models are stacked as 3D to feed into models. Track geometry measurements are collected by TGCs from 2016–2019 and the length of the section is 30 km. Time-series data in terms of time, distance, and maintenance activities are used as features. The study presents the process of developing the 6D BIM model to integrate with machine learning. Initially, 3D BIM models are developed. Then, cost and quantity, schedule, and maintenance information are added to the BIM model to achieve the goal of 4D, 5D, and 6D, respectively.

From the machine learning model development, the performances of developed models are satisfied. The average R^2^ and MAE are 0.95 and 0.56 mm., respectively, which can overcome the state-of-the-art performances. Moreover, this study proposes models that can predict each track geometry parameter. The study demonstrates the potential of the integration between BIM and machine learning for predictive maintenance. In addition, it is practical and improves the overall maintenance performance in the railway industry.

A limitation of the stud is some railway operators might not have data in the format proposed in the study. However, the dimension of inputs can be adjusted in the machine learning model codes. For further study and improvement, more data should be acquired to test whether models still well perform when the sequence of data is longer. Moreover, the length of prediction or window concept can be tested whether predictions can be used to predict further parameters. Other features can be added and explored for influence on the track geometry parameters. More advanced machine learning models should experiment with whether they can provide better performance than the models used in this study.

## Figures and Tables

**Table 1:** Example of responses based on track geometry by TMC 203 [10].

Longitudinal Level	Track Speed (Normal/Passenger) km/h					
	20/20	40/40	60/60	80/90	100/115	115/160
0–16	N	N	N	N	N	N
17–20	N	N	N	N	P3	P2
21–24	N	N	N	P3	P2	P1
25–27	N	N	P3	P2	P1	E2
28–30	N	P3	P2	P1	E2	E2
31–32	P2	P2	P1	E2	E2	E2
33–34	P1	P1	E2	E2	E2	E1
35–40	E2	E2	E2	E2	E1	E1
>40	E1	E1	E1	E1	E1	E1

**Table 2:** Definition of responses [10].

Responses Category	Inspect and Verify Responses	Action
Emergency 1 (E1)	Prior to passage of next train	Prior to passage of next train
Emergency 2 (E2)	Within 2 h. or before the next train, whichever is the greater	Within 24 h
Priority 1 (P1)	Within 24 h.	Within 7 days
Priority 2 (P2)	Within 7 days	Within 28 days
Priority 3 (P3)	Within 28 days	Program for repair
Normal (N)	Nil	Routine inspection

**Table 3:** Summary of previous studies.

Year	Predicted/Measured Parameter	Input	Performance	Limitation	Reference
2019	Longitudinal level and alignment	ABA	R^2^ = 0.63	Only parameters were predicted	Ágh [12]
2006	Vehicle response such as loads	Track geometry and speed	Acceptable	The benefit of railway maintenance was limited	Li, Meddah, Hass and Kalay [13]
2014	Track geometry	ABA	Error = 3 mm.	Each parameter could not be measured	Tsunashima, Naganuma and Kobayashi [14]
2020	TQI	Time-series data	R^2^ = 0.84	Only alignment and longitudinal level were considered	Lee, Hwang, Choi and Choi [15]
2016	Longitudinal level, superelevation, and dip	Track class, traffic volume, and time interval	Accuracy = 0.81	Only three parameters were considered	Hu and Liu [16]
2014	Deterioration	Track structure, traffic characteristics, track layout, environmental factors, track geometry, and maintenance	R^2^ = 0.77	Limited data and samples	Guler [8]
2020	Occurrence of longitudinal defects	speed, TQI, defect, and types of assets	Sensitivity = 0.89	Only one parameter was considered and no regression	Soleimanmeigouni, Ahmadi, Nissen, Xiao and Engineering [17]

**Table 4:** Tuned hyperparameter of each model.

Models	Tuned Hyperparameters	
RNN	Normalization	Batch size
	Number of RNN nodes	Number of dense layers
	Number of hidden nodes	Activation functions
	Optimizer	
LSTM	Normalization	Batch size
	Number of LSTM nodes	Number of dense layers
	Number of hidden nodes	Activation functions
	Optimizer	
GRU	Normalization	Batch size
	Number of GRU nodes	Number of dense layers
	Number of hidden nodes	Activation functions
	Optimizer	

**Table 5:** The best performance to predict each track geometry parameter.

Track Geometry Parameters	Models	MAE (mm)	R^2^	Maximum Absolute Error (mm)	Track Construction Tolerance (mm) [31]
Superelevation	Attention	1.46	0.99	5.00	5.00
Longitudinal level (right rail)	GRU	0.28	0.96	11.26	12.00
Longitudinal level (left rail)	GRU	0.33	0.95	10.06	12.00
Alignment (right rail)	LSTM	0.13	0.98	5.61	7.00
Alignment (left rail)	LSTM	0.20	0.97	6.66	7.00
Gauge	LSTM	1.20	0.84	5.99	6.00
Twist	LSTM	0.33	0.93	5.60	6.00

**Table 6:** Tuned hyperparameters that provide the best performance.

Models	Tuned Hyperparameters	Tuned Values
RNN	Normalization	No
	Batch size	32
	Number of RNN nodes	200
	Number of dense layers	5
	Number of hidden layers	3
	Activation functions	Sigmoid
	Optimizer	Adam
LSTM	Normalization	No
	Batch size	8
	Number of LSTM nodes	3
	Number of hidden layers	2
	Number of hidden nodes	100
	Activation functions	ReLu (dense1) and Linear (dense2 and dense3)
	Optimizer	Adam
GRU	Normalization	No
	Batch size	64
	Number of GRU nodes	3
	Number of hidden layers	2
	Number of hidden nodes	100
	Activation functions	ReLu (dense1 and dense2) and Linear (dense3)
	Optimizer	Adam
Attention	Normalization	No
	Batch size	64
	Type of attention	Self-attention
	Attention activation functions	ReLu
	Number of hidden layers	2
	Number of hidden nodes	100
	Activation functions	ReLu (dense1 and dense2) and Linear (dense3)
	Optimizer	Adam

## Data Availability

The data presented in this study are available on request from the corresponding author.
